# Donor impurity energy and optical absorption in spherical sector quantum dots

**DOI:** 10.1016/j.heliyon.2020.e03194

**Published:** 2020-01-17

**Authors:** M.E. Mora-Ramos, A. El Aouami, E. Feddi, A. Radu, R.L. Restrepo, J.A. Vinasco, A.L. Morales, C.A. Duque

**Affiliations:** a Centro de Investigación en Ciencias, Instituto de Investigación en Ciencias Básicas y Aplicadas, Universidad Autónoma del Estado de Morelos, Av. Universidad 1001, CP 62209, Cuernavaca, Morelos, Mexico; b Grupo de Materia Condensada-UdeA, Instituto de Física, Facultad de Ciencias Exactas y Naturales, Universidad de Antioquia UdeA, Calle 70 No. 52-21, Medellín, Colombia; c Laboratoire de Matiére Condenssée et Sciences Interdisciplinaires (LaMCScI), Group of Optoelectronic of Semiconductors and Nanomaterials, ENSET, Mohammed V University in Rabat, Morocco; d Department of Physics, “Politehnica” University of Bucharest, 313 Splaiul Independenţei, Bucharest, RO-060042, Romania; e Universidad EIA, CP 055428, Envigado, Colombia

**Keywords:** Condensed Matter Physics, Nanotechnology, Quantum dot, Donor impurity, Binding energy, Optical Absorption

## Abstract

The properties of the conduction band energy states of an electron interacting with a donor impurity center in spherical sector-shaped GaAs-Al_0.3_Ga_0.7_As quantum dots are theoretically investigated. The study is performed within the framework of the effective mass approximation through the numerical solution of the 3D Schrödinger equation for the envelope function via the finite element method. The modifications undergone by the spectrum due to the changes in the conical structure geometry (radius and apical angle) as well as in the position of the donor atom are discussed. With the information regarding electron states the linear optical absorption coefficient associated with transition between confined energy levels is evaluated and its features are discussed. The comparison of results obtained within the considered model with available experimental data in GaAs truncated-whisker-like quantum dots shows very good agreement. Besides, our simulation leads to identify the lowest energy photoluminescence peak as donor-related, instead of being associated to acceptor atoms, as claimed after experimental measurement (Hiruma et al. (1995) [14]). Also, a checking of our numerical approach is performed by comparing with analytical solutions to the problem of a spherical cone-shaped GaN with infinite confinement and donor impurity located at the cone apex. Coincidence is found to be remarkable.

## 1 Introduction

Quantum dots (QDs) are crystalline solid structures of nanoscopic dimensions that can be considered as quasi-zero-dimensional electronic systems, since the motion of charge carriers in them are constrained to have only well-defined energy values. Such a discrete spectrum has led some authors to name these nanosystems as “artificial atoms”. They are mainly made of semiconductor materials and have found broad application in distinct areas of technology and science, including medicine. Recent advances in the area of QDs are reviewed in Refs. [1], [2], [3], [4].

The study of semiconductor QDs has included different geometries for these structures: spherical, lens-shaped, and pyramidal. The cone-like QDs have also been investigated and their electronic and optical properties, including the effects of donor impurities, electric and magnetic fields, have been reported by several authors [5], [6], [7], [8], [9], [10], [11], [12], [13]. It is worth noting the experimental realization of microcrystals and conical-shaped heterostructures reported in the works of Hiruma et al. [14] and Schamp et al. [15]. The potential applications of these GaAs whiskers for light emitting devices is a source of permanent research because a semiconductor wire structure employing quantum size effects is a very important element of electronic and optical devices.

Modeling of different properties of semiconductor QDs using the finite element method (FEM) can be traced back to early nineties (see, for instance the work of Ref. [16]). Posteriorly, and also in recent years, it is possible to mention a number of studies dealing with structural, electronic and optical behaviors, transport, impurity, and strain effects in dot-like nanosystems of distinct shapes and composition [17], [18], [19], [20], [21], [22], [23], [24], [25], [26], [27]. A general environment that combines k⋅p and FEM methods for band structure calculation in nanostructures has been put forward by Veprek and collaborators [28].

In the present work we investigate the electronic and optical properties of conical quantum dots with spherical upper cap [abbreviated as spherical cone-shaped (SCS) QD]. It is assumed that a donor impurity center is located somewhere along the vertical axis of the cone. The allowed electron states in the structure are determined through the numerical solution of the effective mass equation for the envelope function under the FEM approach. The coefficient of inter-level optical absorption is then evaluated as a function of the incident light frequency for different geometric configurations of the SCS-QD. The article has the following organization: The description of the theoretical framework is presented in section 2. The section 3 contains a discussion of the properties of electronic levels and the intraband optical response. Finally, in the section 4 we outline the conclusions.

## 2 Theoretical framework

**Figure 1:**
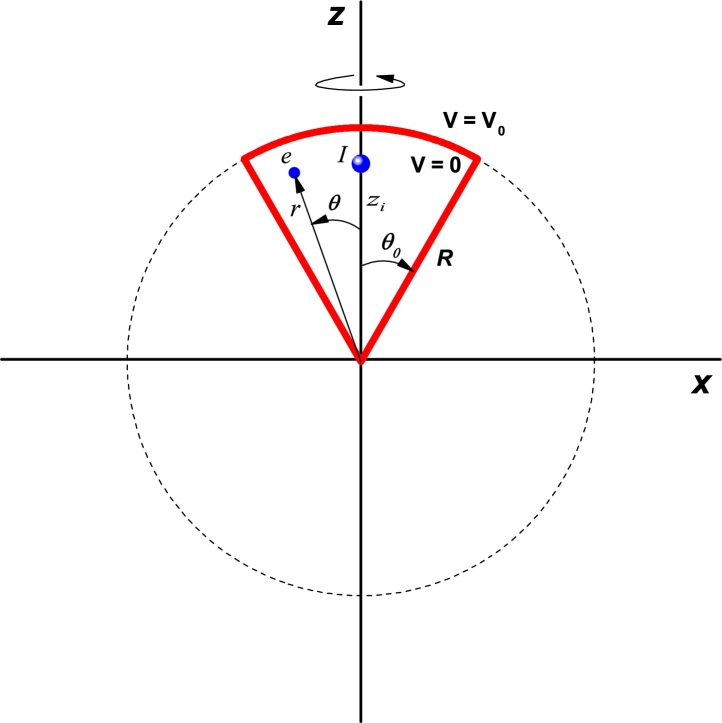
Pictorial view of the *y* = 0 projection of a spherical cone-shaped quantum dot. The dimensions of the structure are the radius (*R*) and the apical angle (*θ*_0_). A donor impurity has been considered with its axial coordinate *z*_*i*_. The spherical coordinates of the electron are (*r*,*θ*,*φ*). The confinement potential is defined as zero inside the quantum dot structure and *V*_0_ elsewhere.

The type of QD considered here has a conical shape with spherical upper cap as can be schematically seen in the Fig. 1. We are assuming that the conduction band profile of the structure incorporates a finite height potential energy well ($V_{0}$) that can be practically achieved by embedding the dot system within a matrix of a larger gap material. Without a significant loss of generality, the ionized donor atom is taken to be located on the vertical cone axis, with the origin of coordinates coinciding with the cone vertex.

**Figure 2:**
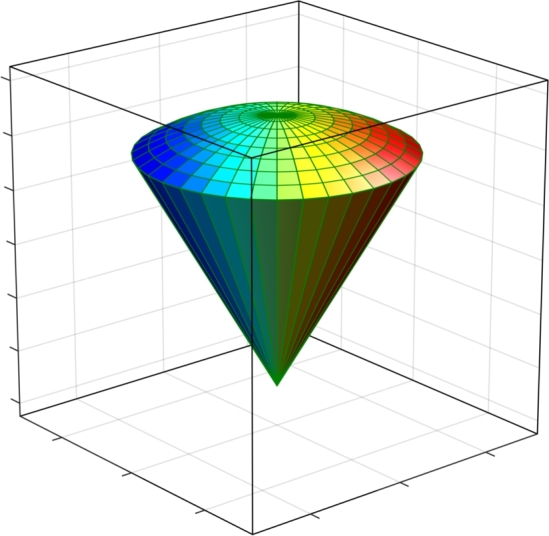
Pictorial view of the 3D-quantum dot structure inside a 3D cubic box. The Dirichlet boundary conditions establish that the wave function is zero on the six faces of the cubic region.

The allowed electron states are obtained numerically by solving the 3D conduction-band-effective-mass Schrödinger equation for the smoothly varying envelope wave function, with confining potential equal to zero inside the cone region and to $V_{0}=const.$ outside. In addition, the differential equation includes a Coulombic potential term representing the attractive interaction between the electron and the ionized donor impurity center. We are taking into account the dependence of the electron effective mass with position, having constant but distinct values on both sides of the QD's surface. This implies that the Ben-Daniel-Duke type matching conditions will have to be imposed over it. The calculation process is carried out using the FEM, as implemented in the commercially available COMSOL Multiphysics software [29]. As usual, the binding energy of the electron to the impurity center is determined by subtracting the result obtained when the Coulomb interaction is present from the electron energy in the conduction band without the electrostatic coupling. In the Fig. 2 we represent a pictorial view of the spatial setup used in the simulation, considering Dirichlet boundary conditions at long enough distance from the active QD region. In our particular case, the material inside the QD is GaAs whilst the “barrier” region is taken to be made of Al_0.3_Ga_0.7_As.

Summarizing then all the points of the previous paragraph, the problem to be solved corresponds to an electron confined in a conical region of *R*-radius and $\theta_{0}$-apical angle with V(x,y,z)-confinement potentials whose values are zero in the inner region of the cone, $V_{0}$ in the region surrounding the quantum dot, and infinity in the outer region of a parallelepiped (see Fig. 2). The dimensions of the outer box are large enough so that it can be considered that there are no confinement effects on the carriers. Taking into account the presence of a donor impurity located on the *z*-axis, the Schrödinger equation of the system is given by

(1) $$[-\frac{\hbar^{2}}{2}\;\nabla \cdot \left(\frac{1}{m^{⁎}(x,y,z)}\;\nabla \right)+V(x,y,z)\;-\frac{e^{2}}{4\pi \varepsilon_{0}\varepsilon_{r}\;\sqrt{x^{2}+y^{2}+{\left(z-z_{i}\right)}^{2}}}]\;\Psi (x,y,z)=E\;\Psi (x,y,z)\;,$$

where $z_{i}$ represents the impurity position along the *z*-axis, $\varepsilon_{r}=13.0$ is the GaAs static dielectric constant, and $m^{⁎}(x,y,z)$ is the position dependent effective mass which value is $0.0665\;m_{0}$/$0.092\;m_{0}$ in the dot/barrier region (where $m_{0}$ is the free electron mass). The confinement potential equals zero inside the GaAs dot and 262 meV in the Al_0.3_Ga_0.7_As surrounding material.

The solution of Eq. (1) consists in finding the Ψ(x,y,z) wave functions and their corresponding energies. This is a problem with an exact analytical solution in those cases where the confinement potential outside the cone region is infinite and the impurity is located at its apex (see the details below in subsection 2.1). To proceed with the current problem, it is necessary to resort to numerical methods, which in our case makes use of the finite element technique (see the details below in subsection 2.2).

To include the study of the inter-level optical absorption response we calculate the respective coefficients using the following expression [30], [31], [32]:

(2) $$\alpha_{fi}(\omega )=\omega \;\sqrt{\frac{\mu }{\varepsilon_{R}}}\;\frac{\sigma \;\hbar \;\Gamma_{fi}|{\overset{˜}{M}}_{fi}{|}^{2}}{{\left(E_{f}-E_{i}-\hbar \;\omega \right)}^{2}+{\left(\hbar \;\Gamma_{fi}\right)}^{2}}\;,$$

where $\sigma =3.0\times 10^{22}{\text{ m}}^{-3}$ is the electron density and the energy-conserving Dirac delta function $\delta (E_{f}-E_{i}-\hbar \;\omega )$ has been replaced by a Lorentzian factor, that includes a phenomenological damping term, $\Gamma_{fi}=1.5$ THz, related with |i〉→|f〉 transition lifetimes. In the former equation *ω* is the frequency of the incident photon, *μ* is the vacuum magnetic permeability, and $\varepsilon_{R}=\varepsilon_{0}\;\varepsilon_{r}$. The quantity ${\overset{˜}{M}}_{fi}=\langle i|e\;\xi |f\rangle$ is the transition electric dipole moment matrix element between the initial and final states involved, *e* being the electron charge. In the present work, two polarizations of the incident radiation have been considered: *i*) linearly-polarized light, with ξ=z, and *ii*) circular-polarized light, with $\xi =(x\pm i\;y)/\sqrt{2}\equiv +$.

### 2.1 Analytical solution for on-corner donor impurity in spherical sector quantum dots with infinite confinement potential

In this subsection we will discuss the analytical solution for wave functions and their corresponding energies of a donor impurity placed at the apex of a spherical sector quantum dot (coinciding with the origin of coordinates) with infinite confinement potential. Considering spherical coordinates (r,θ,φ) and using the effective Bohr radius $\left(a^{⁎}=\frac{\hbar^{2}\epsilon }{m_{e}^{⁎}e^{2}}\right)$ as unit of length and the effective Rydberg $\left(R^{⁎}=\frac{e^{2}}{2\varepsilon {\left(a^{⁎}\right)}^{2}}\right)$ as unit of energy, the Hamiltonian of the problem in the effective mass approximation can be written in the form

(3) $$\{-\left[\frac{1}{r^{2}}\frac{\partial }{\partial r}\left(r^{2}\frac{\partial }{\partial r}\right)+\frac{1}{r^{2}\sin\theta }\frac{\partial }{\partial \theta }\left(\sin\theta \frac{\partial }{\partial \theta }\right)+\frac{1}{r^{2}\sin^{2}\theta }\frac{\partial^{2}}{\partial \varphi^{2}}\right]\;-\frac{2}{r}+V_{W}\}\Psi_{D}(r,\theta ,\varphi )=E_{D}\Psi_{D}(r,\theta ,\varphi )\;,$$

where $m_{e}^{⁎}$ represents the conduction effective mass and *ε* corresponds to the static dielectric constant. The corresponding confinement potential is, in this case:

(4) $$V_{W}=\left\{ \begin{array}{cc} 0\; & \;\;\;\text{if }\;\;\;r<R\text{ and }\theta <\theta_{0}\\ \infty \; & \;\;\text{otherwhere}\;, \end{array}\right.$$

thus implying that the probability density for an electron outside the structure is zero. Taking into account: *i*) that the impurity is located at the apex of the cone, which implies that the problem has azimuthal symmetry around the *z*-axis (φ=0) and *ii*) the infinite confinement potential considered in this part, then the wave function in Eq. (3) can be written in the form

(5) $$\Psi_{D}(r,\theta ,\varphi )=N\;ℜ(r)\;\Theta (\theta )\;\;e^{im\varphi }\;,$$

where *m* is an integer number, $i^{2}=-1$, and *N* is a normalization constant. Replacing the Eq. (5) into the Eq. (3), it is possible to obtain two independent differential equations:

(6) $$\frac{1}{\sin\theta }\frac{d}{d\theta }\left(\sin\theta \frac{d\Theta (\theta )}{d\theta }\right)+\left(\nu^{2}-\frac{m^{2}}{\sin^{2}\theta }\right)\Theta (\theta )=0$$

and

(7) $$\frac{d}{dr}\left(r^{2}\frac{dℜ(r)}{dr}\right)+(k^{2}r^{2}-\nu^{2})ℜ(r)-2\;r\;ℜ(r)=0\;,$$

where $k=\sqrt{E_{D}}$.

If we choose the special case of m=0 in Eq. (5), then the solutions of Eq. (6) are linear combinations of the Legendre polynomials of first and second kinds $P_{\nu }(\cos\theta )$ and $Q_{\nu }(\cos\theta )$, respectively:

(8) $$\Theta (\theta )=C_{1}P_{\nu }(\cos\theta )+C_{2}Q_{\nu }(\cos\theta )\;.$$

It is determined by imposing the nullity of the wave function at the geometrical limit of the dot ($\theta =\theta_{0}$). It turns out that $Q_{\nu }(x)$ diverges when *x* tends to 1. So, the Θ(θ) wave function reduces to:

(9) $$\Theta (\theta )=C_{1}P_{\nu }(\cos\theta )\;.$$

The solution for the Eq. (7) is a linear combination of Whittaker functions of first and second kinds: $M_{t,\nu +1/2}(2ikr)$ and $W_{t,\nu +1/2}(2ikr)$, respectively

(10) $$ℜ(r)=\frac{1}{r}\left[C_{3}M_{t,\nu +1/2}(2ikr)+C_{4}W_{t,\nu +1/2}(2ikr)\right]\;,$$

where $t=-\frac{i}{E_{D}}$. We note that $W_{t,\nu +1/2}(2ikr)$ diverges when r→0, so we choose $C_{4}=0$. Thus, the expression for the wave function actually reduces to:

(11) $$\Psi_{D}(r,\theta )=\frac{N}{r}M_{t,\nu +1/2}(2ikr)\;P_{\nu }(cos\;\theta )e^{im\varphi }\;.$$

The procedure is as follows: *i*) in Eq. (9) the values of *ν* are obtained by imposing the condition $P_{\nu }(\cos\;\theta_{0})=0$ and *ii*) the values obtained for *ν* in the previous step are replaced in Eq. (10), where $C_{4}=0$, in order to obtain the energies associated with each value of *ν* by using the condition $M_{t,\nu +1/2}(2ikR)=0$. Note that the *k* and *t* parameters depend on the energy $E_{D}$.

### 2.2 The finite element method

The FEM is a very powerful numerical technique for solving partial differential equations (PDEs) defined in complex or irregular geometry domains. Hence, the Schrödinger equation is a candidate to be solved by this method since the cases in which an analytical solution is available are very limited and with respect to the shape, real structures usually show not regular or very complex geometries. This method consists of dividing a problem from the continuous domain into a large but finite set of algebraic equations each being defined in a small domain called the finite element.

The weak formulation is used in conjunction with the discretization of the PDE. This consists of transforming the PDE from its differential operatorial form into an integral form, and in terms of discretization the integrals are replaced by sums. In systems formed by two or more materials, the effective mass is not a constant in the whole structure. Hence, a more general form of the Schrödinger equation for a potential *V* is

(12) $$\nabla \cdot \left[\left(-\frac{\hbar^{2}}{2m^{⁎}}\right)\nabla \right]\psi +V\psi =E\psi \;,$$

where *ħ* is the reduced Planck's constant, $m^{⁎}$ is the carrier effective mass, *ψ* the wave function, and *E* the energy. Multiplying this equation by some test function *ϕ*, integrating in the volume Ω, and by using the property $\nabla \cdot (\overset{\to }{F}v)=\overset{\to }{F}\cdot \nabla v+(\nabla \cdot \overset{\to }{F})v$, where $\overset{\to }{F}=-\frac{\hbar^{2}}{2m^{⁎}}\nabla \psi$ and v=ϕ, the Eq. (12) becomes

(13) $$\int_{\Omega }\frac{\hbar^{2}}{2m^{⁎}}\nabla \psi \cdot \nabla \phi \;d\Omega +\int_{\Omega }\nabla \cdot \left[\left(-\frac{\hbar^{2}}{2m^{⁎}}\nabla \psi \right)\phi \right]\;d\Omega +\int_{\Omega }(V-E)\psi \;\phi \;d\Omega =0\;.$$

Using the Green's theorem and the flow condition for a stationary problem, it is easy to prove that the second term on the left side in Eq. (13) is zero. Consequently, that expression is finally transformed into:

(14) $$\int_{\Omega }\frac{\hbar^{2}}{2m^{⁎}}\nabla \psi \cdot \nabla \phi \;d\Omega +\int_{\Omega }(V-E)\psi \;\phi \;d\Omega =0\;.$$

Note that Eq. (14) includes the overlap either of functions or gradient of them, which simplifies the calculations to small regions where the overlap is not zero. The great advantage of the weak form is that no second order derivative is involved and the functions *ψ* and *ϕ* have to be continuous and differentiable only in subdomains.

**Figure 3:**
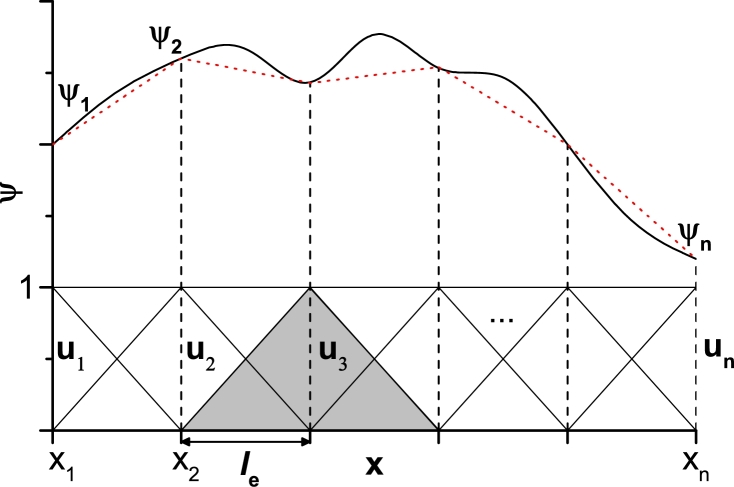
Illustration of the FEM discretization. The *u*_*n*_ are linear base functions and *ψ*_*n*_ are the coefficients that adjust the numerical solution to the exact solution.

The Fig. 3 illustrates how a domain is discretized in order to use the FEM. In this example, the function *ψ* has been divided into n−1 subdomains (elements) with the same length $l_{e}$ (regular partition), which corresponds to *n* nodes. The red dotted line is the approximation by FEM of the exact values illustrated with solid line. Inside a subdomain, delimited by the vertical black dotted lines, only two base functions contribute, the others being zero. In the figure is used a base of linear functions $u_{i}$, i=1,..,n with a value of 1 for its respective node and 0 in any other node. Therefore, the function *ψ* can be approximated by the linear combination $\psi =\sum_{i}u_{i}\psi_{i}$. Note that the coefficients $\psi_{i}$ are exact solutions in the respective *x*−values of the nodes because $u_{i}$ is 1 there. The coefficients $\psi_{i}$ are obtained from the equation that governs the problem under study, simplified as a set of algebraic equations due to the discretization.

There are two common ways to choose the functions in the discretization, the Rayleigh-Ritz and Galerkin methods [33]. The main difference between the two is that Galerkin method uses the same set of functions as the base and test functions. In practice, the discretization is obtained by turning the PDE into the weak form. The integrals that appear in the transformation are evaluated within a single element and a representation in the whole domain of the equation corresponds to the sum of each partition contribution. This results in a system of *n* algebraic equations corresponding to the n−1 partitions (*n* nodes).

In Fig. 3, the shaded base function represents the periodic behavior in a regular partition. Taking into account that the PDE weak form contains the multiplication of the solution and the base functions or their gradients, the integral overlapping only occurs between two neighboring functions, which facilitates the calculations. One of the advantages of using this method is that irregular partitions can be made, for example, in those regions where the gradient is large, smaller elements can be built. Another advantage is the possibility of using several base functions adapting to the problem under study.

In the next section we present and discuss our results for the energy spectrum, the binding energy of the ground state, the expected values of the dipole moments, and optical absorption for a confined electron in a SCS-QD, all this considering multiple variants of the heterostructure dimensions (radius and apical angle) and impurity position.

## 3 Results and discussion

**Figure 4:**
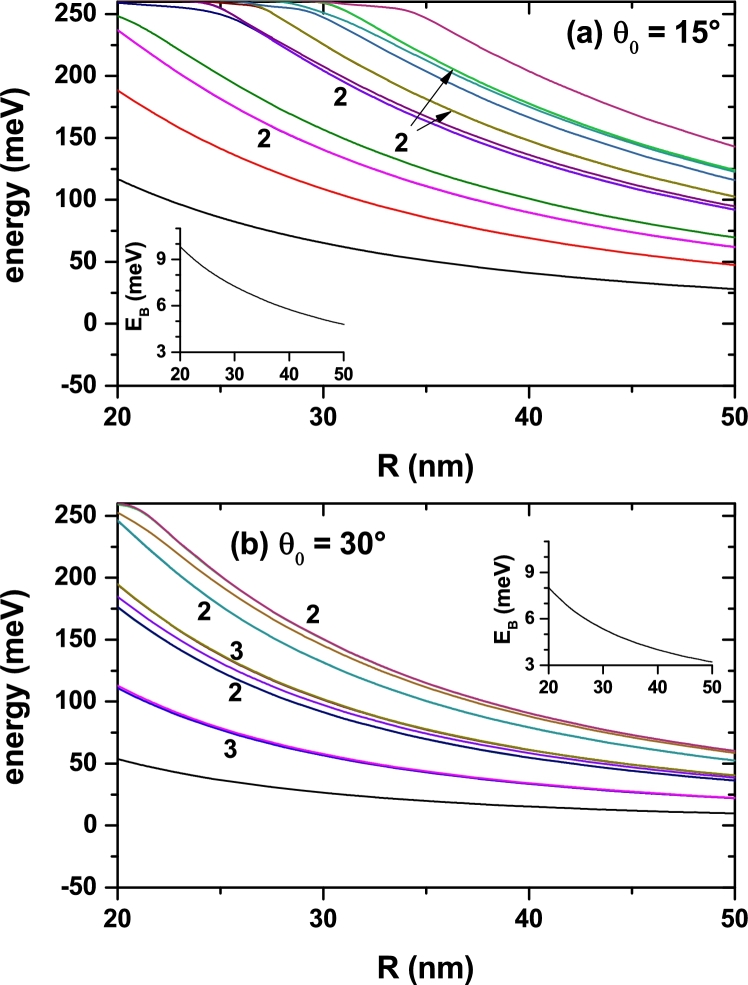
Energy of the lowest fifteen energy levels for a confined electron in a spherical cone-shaped GaAs-Al_0.3_Ga_0.7_As quantum dot as a function of the structure radius. The results are for *z*_*i*_ = 0 with *θ*_0_ = 15° (a) and *θ*_0_ = 30° (b). Labels 2 and 3 indicate doubly or triple degenerate states. The insets show the binding energy for the ground state.

We are interested in studying the effect of the impurity position. In accordance, the calculation started by putting the donor atom at the very vertex of the conical QD. The results obtained for the lowest confined state energies are depicted in Fig. 4 as functions of the cone size (radius, *R*, see Fig. 1), with two different values of the apical angle, $\theta_{0}$. It can be noticed that for a small value $\theta_{0}=15{}^{\circ}$ (Fig. 4(a)), and in the entire range of considered radii, the system supports at least five states confined in the cone. Note that for R=20 nm, the fifth state has slightly less energy than the height of the potential barrier, $V_{0}=262$ meV. For R>35 nm, the fifteen reported states are confined in the cone. Besides, the energy of the higher excited levels for small radii approaches the upper bound posed by the potential barrier height and starts having a decreasing variation when the dot's size becomes large enough. Eventually, all levels will decrease with the increment of *R*, but excited states with higher energies will do that in a slower manner than for the case in Fig. 4(b). Clearly, it is a consequence of the greater electron spatial localization associated to a stronger confinement when the apical angle is small. The contrasting situation is shown in Fig. 4(b). One may observe that when $\theta_{0}=30{}^{\circ}$ there are already fifteen energy levels for R=20 nm, and the rate of energy reduction is larger when the radius augments. It is worth bringing the reader's attention to the fact that we are depicting the first fifteen electron levels, including degenerate ones. So, although only ten curves are seen, all energies are actually shown. The labels 2 and 3 in Fig. 4 indicate the states degeneration degree that have been shown.

With regard to the impurity binding energy, $E_{b}=E_{0}-E$ (where $E_{0}$/*E* represents the eigenvalues of the Eq. (1) without/with the Coulomb term), the insets in Figs. 4(a, b) present only the case associated with the ground state. There it is observed a slight increase in this quantity mainly for the narrower conical dot (the binding energy curve in Fig. 4(a) is blueshifted with respect to the corresponding results in Fig. 4(b)). For large values of the apical angle, in Fig. 4(b), the wave functions of the uncorrelated electrons tend to be located near the cone gravity center, that is, at an average distance to the impurity of 2/3 of the cone height (note that in this case the impurity is located at the vertex of the structure). By decreasing the cone angle, Fig. 4(a), two effects appear: *i*) by the presence of lateral potential barriers, the confinement effect on the carriers increases, and consequently their kinetic energy, and *ii*) the system tends to approximate a 1D quantum wire, where the wave functions are distributed regularly along the wire. This second effect translates into an effective decrease in electron-impurity distance, which, combined with the increase in geometric confinement, ultimately leads to larger binding energy. The reduction of the electron binding energy for greater values of *R* is related to the increase in the effective electron-impurity distance due to the enlargement of SCS-QD size.

**Figure 5:**
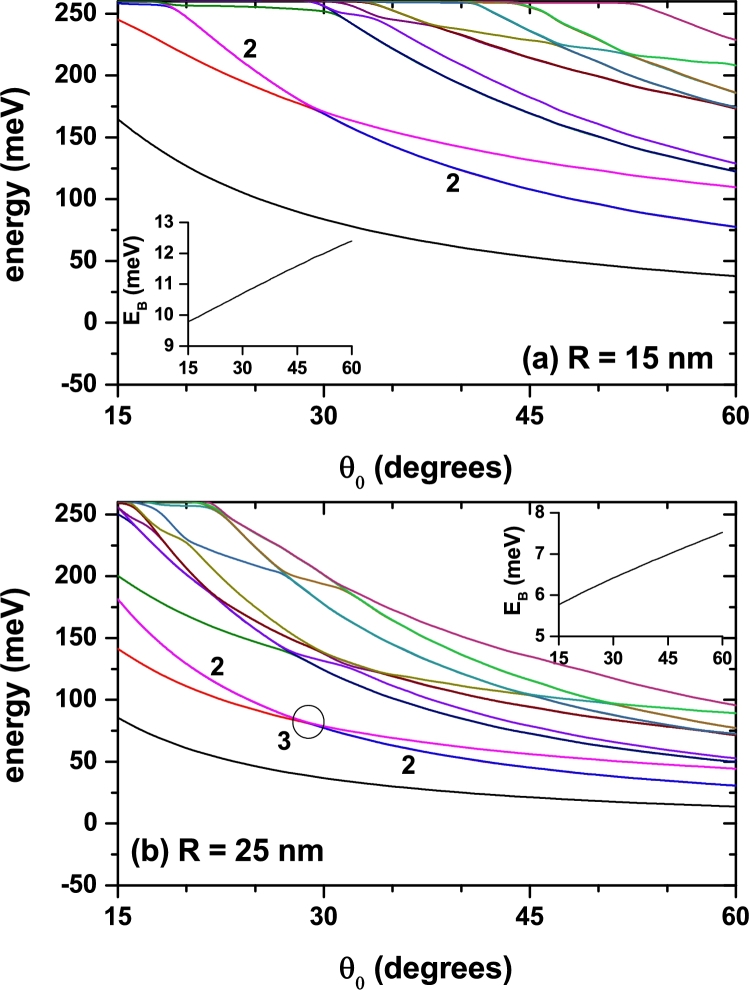
Energy of the lowest fifteen energy levels for a confined electron in a spherical cone-shaped GaAs-Al_0.3_Ga_0.7_As quantum dot as a function of the apical angle of the structure. The results are for *z*_*i*_ = 0 with *R* = 15 nm (a) and *R* = 25 nm (b). Labels 2 and 3 indicate doubly and triple degenerate states. The circle in panel (b), localized at *θ*_0_ = 29° with *E* = 79 meV, indicates the presence of the accidentally triply degenerate states, as depicted in Fig. 4(b). The insets show the binding energy for the ground state.

An analogous situation for the electron energies in the SCS-QD can be observed in Fig. 5 in which two cases of fixed dot's radius and varying apical angle are shown. The changes in the allowed levels can be, again, explained by the analysis of spatial electron confinement, provided that augmenting the apical angle implies the enlargement of the GaAs region size. However, in this case it is possible to note that at certain values of $\theta_{0}$ there are crossings between some degenerate excited states. The more noticeable feature is that the general behavior of the ground state binding energy is now an increasing one. The physical reason behind this phenomenon is the reduction of the effective electron-impurity distance resulting from the increment of the spatial localization of the ground state probability density in the region adjacent to the cone vertex, where the impurity is placed. In fact, because the range of geometric parameters considered, the wider the angle, the greater will be the spatial confinement of the ground state wave function around the vertex.

With respect to the results shown in Figs. 4 and 5, it is important to say that when R→0 ($\theta_{0}\to 0$) in Fig. 4 (in Fig. 5) and without impurity effects, the energies of the system tend to the exact spectrum values of a quantum box with infinite confinement potential, whose solutions are analytical and very well-known from the literature. In that case, the spectrum of the box has as reference energy the value of $V_{0}=262$ meV (which is the value used in this study). When considering the effects of an impurity, in the limit in which the volume of the cone tends to zero (i.e. when R→0 or $\theta_{0}\to 0$), the energy spectrum corresponds to the solution of an impurity confined inside a box with infinite confinement. Due to the fact that they are outside the cone region, such solutions are not of interest for the study reported in this article. Our studies have shown that as the cone dimensions tend to zero the system has a binding energy for the ground state of 1.5 effective Rydbergs, which is slightly greater than the value of 1 effective Rydberg corresponding to hydrogenic atoms in the bulk. This value demonstrates a confinement effect associated with the large box (see Fig. 2) used in the numerical process to obtain solutions of the eigenvalues differential equation. The confinement effect of the large box is imperceptible in the results reported in this study since for the considered dimensions more than 95% of the wave functions of the reported states are confined in the cone region. It is important to say that in the study the convergence of energies is down to 0.1 meV for the first 15 confined states.

**Figure 6:**
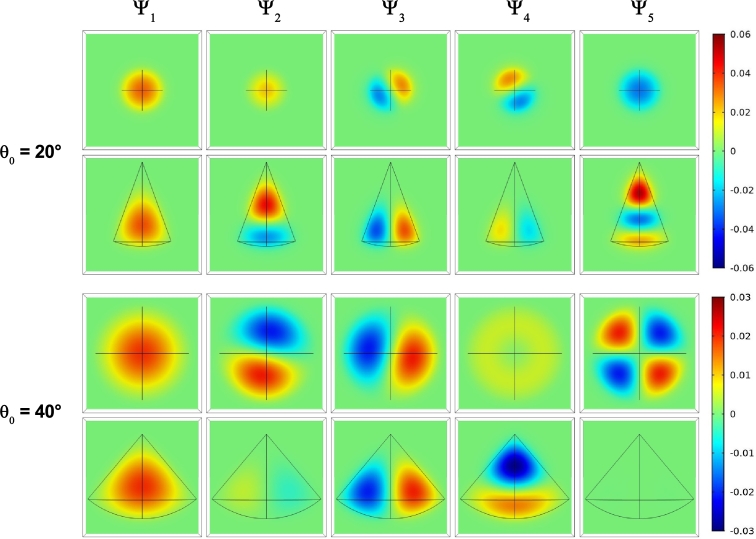
The *z* = 20 nm and *y* = 0 projections of the normalized wave function for the five lowest confined electron states in a spherical cone-shaped GaAs-Al_0.3_Ga_0.7_As quantum dot. Results are for *R* = 30 nm with *θ*_0_ = 20° (first row) and *θ*_0_ = 40° (second row). In the figure the color bars corresponding to each of the two considered angles have been included.

To clarify the degenerations and symmetries of the different states reported in Figs. 4 and 5, we proceed to present in Fig. 6 the z=20 nm and y=0 projections of the first five confined state wave functions of an electron in a spherical cone-shaped GaAs-Al_0.3_Ga_0.7_As QD. We have chosen a fixed value of the radius and two different values of the apical angle. The reason for this choice lies in the fact that in Fig. 4 there is no presence of crossings between levels and therefore in the entire range of calculated radii each state retains its symmetry. In the case of Fig. 5, it is clearly observed that for both R=15 nm and R=25 nm a level crossing appears for $\theta_{0}≅29{}^{\circ}$. Therefore, we have decided to choose two angles, one before the crossing and another after the crossing. For both values of the apical angle it is observed that the ground state has *s*-like symmetry deformed along the *z*-axis such that the wave function adjusts to the shape of the structure. In the case of the states $\Psi_{2}$, $\Psi_{3}$, and $\Psi_{4}$ states the *p*-like symmetries are clearly appreciated, but note that while for $\theta_{0}=20{}^{\circ}$ the $p_{z}$-like state is the first excited (not degenerate), in the case of $\theta_{0}=40{}^{\circ}$ it is the third excited one.

At $\theta_{0}=20{}^{\circ}$, the $\Psi_{3}$ and $\Psi_{4}$ states are degenerated. About the $\Psi_{5}$ state, it is observed that for $\theta_{0}=20{}^{\circ}$, it is a state with three anti nodes along the *z*-direction and that it has even symmetry with respect to the x=0 and y=0 planes. For $\theta_{0}=40{}^{\circ}$, the $\Psi_{5}$ wave function has quite different characteristics. First, it is an odd function with respect to the x=0 and y=0 planes and it is approximately an even function with respect to the z=0 plane (note that the wave functions take the shape of the cone). Note that the wave functions that have azimuthal symmetry with respect to the *z*-axis have anti nodes near the impurity, which is located at the vertex of the QD, while the odd states with respect to the x=0 and y=0 planes are located mainly in the spherical region of the structure. The details presented here will be useful for interpreting the matrix elements for different optical transitions considering linear polarizations along the *z*-direction and circular polarization in the *xy*-plane. Finally, note that for both considered angles, the $p_{x}$ and $p_{y}$ states appear rotated with respect to the *z*-axis. This is a phase effect introduced by the calculation method and which requires attention when studying optical properties with linear polarizations of the incident radiation along the *x*- and *y*-axes since some difficulty can be generated by the apparent selection rules that are involved.

Here we must clarify what we understand by $p_{z}$-like states such as those shown for the $\Psi_{2}$ wave function when $\theta_{0}=20{}^{\circ}$ and for the $\Psi_{4}$ wave function when $\theta_{0}=40{}^{\circ}$. In both cases, along the *z*-direction the wave functions have a node (the wave function is null) and two antinodes with opposite signs (two maxima). In a cylindrical structure, due to its symmetry, these two nodes would imply odd symmetry with respect to the point where the wave function is canceled (the node) and the $p_{z}$ assignment is exact. That is not the case in our study given the breaking of symmetry imposed by the conical shape of the quantum dot. That is the reason why the $p_{z}$ assignment to these states is not completely accurate and should be viewed carefully.

**Figure 7:**
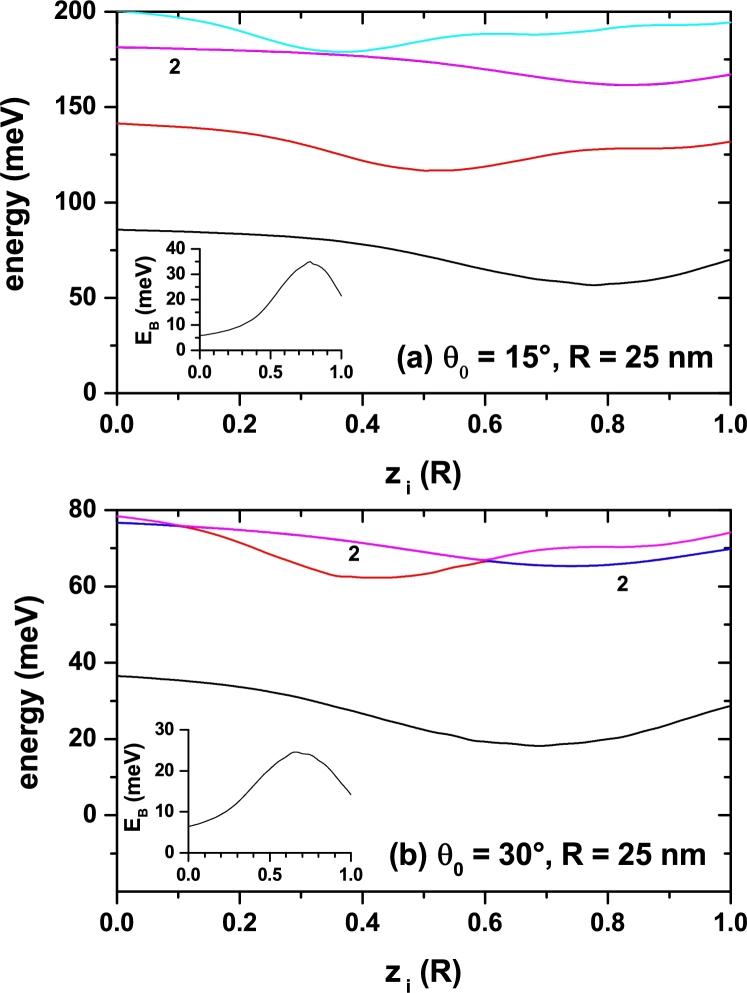
Energy of the lowest states for a confined electron in a spherical cone-shaped GaAs-Al_0.3_Ga_0.7_As quantum dot as a function of the impurity position along the *z*-axis. The results are for *R* = 25 nm with *θ*_0_ = 15° (a) and *θ*_0_ = 30° (b). Label 2 indicates doubly degenerate states. The insets show the binding energy for the ground state.

The displacement of the impurity center location along the symmetry axis produces certain modifications to the electron energies. In order to verify this we have plotted in Fig. 7 the dependence of the lowest level energies with respect to $z_{i}$. Two different geometries with fixed *R* and $\theta_{0}$ are taken into account. It can be noticed that, as expected, the greater energy values correspond to the narrower cone, Fig. 7(a), and that there are some slight oscillations of them with minima occurring at different angular amplitudes for the ground and first two excited states. These minima are associated to the relation between the state symmetry and the cone symmetry. For instance, in the case of the ground state, the position $z_{i}\approx 0.7\;R$ corresponds to a point of maximum axial symmetry of the corresponding probability density. Although it is not accurate, in those places where the ground state has its minimum, we can affirm that the impurity is located near the gravity center of the cone. In Fig. 7(a) it is observed that for $z_{i}=0.34\;R$ a triple degenerate state appears (corresponding to three states with *p*-like symmetry). These three states appear with higher energies than that corresponding to the first excited state, which is clearly associated with confinement along the axial direction. In Fig. 7(b), that triple degenerate state corresponds to the first excited one and appears in $z_{i}=0.6\;R$. It can then be appreciated that the order in which the degenerate states appear with respect to the others that are not, depends on the apical angle, which is finally the one that controls the relative position of the gravity center of the structure. The behavior of the ground state electron-impurity binding energy follows that of the correlated ground level (*E*) but inversely. That is, when the correlated ground state energy has a minimum, $E_{b}$ has a maximum. Such a maximum in the binding energy implies that the effective electron-impurity distance reaches its minimum value. We have to emphasize that for calculations of the insets in Figs. 7(a) and 7(b), the uncorrelated energies are constant functions of $z_{i}$ and only depend on the structure size.

**Figure 8:**
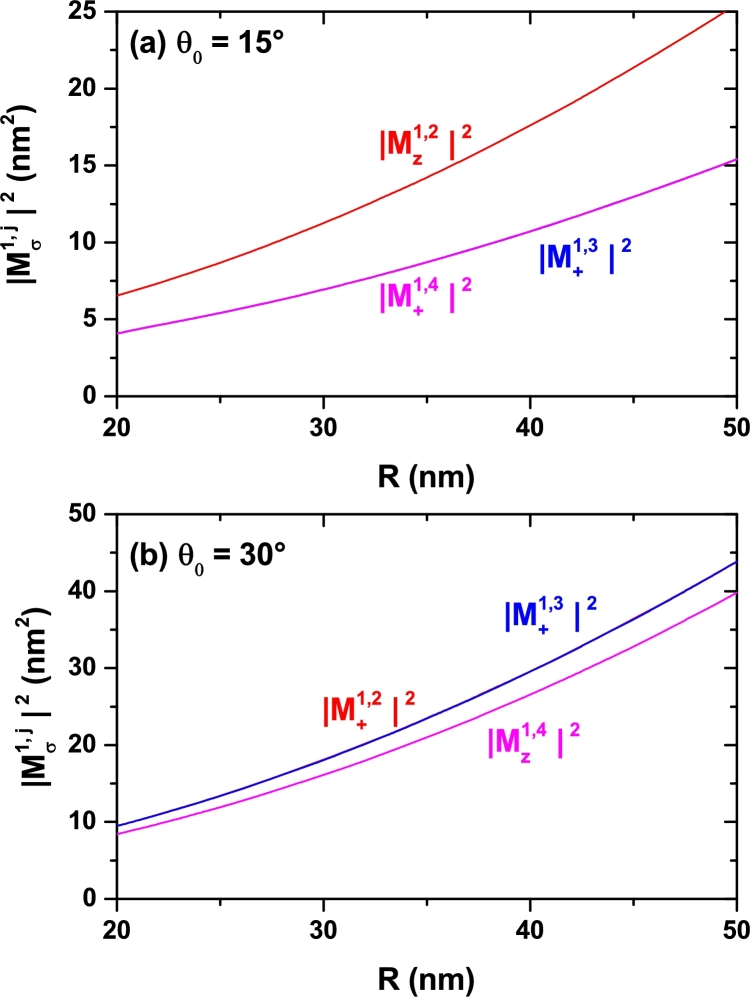
Square of the nondiagonal reduced dipole matrix elements from the ground state to the several lowest excited states (1 → *n*, with *n* = 2,3,4,…) for a confined electron in a spherical cone-shaped GaAs-Al_0.3_Ga_0.7_As quantum dot as a function of the structure radius. Calculations are for *z*-polarized (*ξ* = *z*) and circular-polarized (*ξ* = +) incident radiation. The results are for *z*_*i*_ = 0 with *θ*_0_ = 15° (a) and *θ*_0_ = 30° (b).

**Figure 9:**
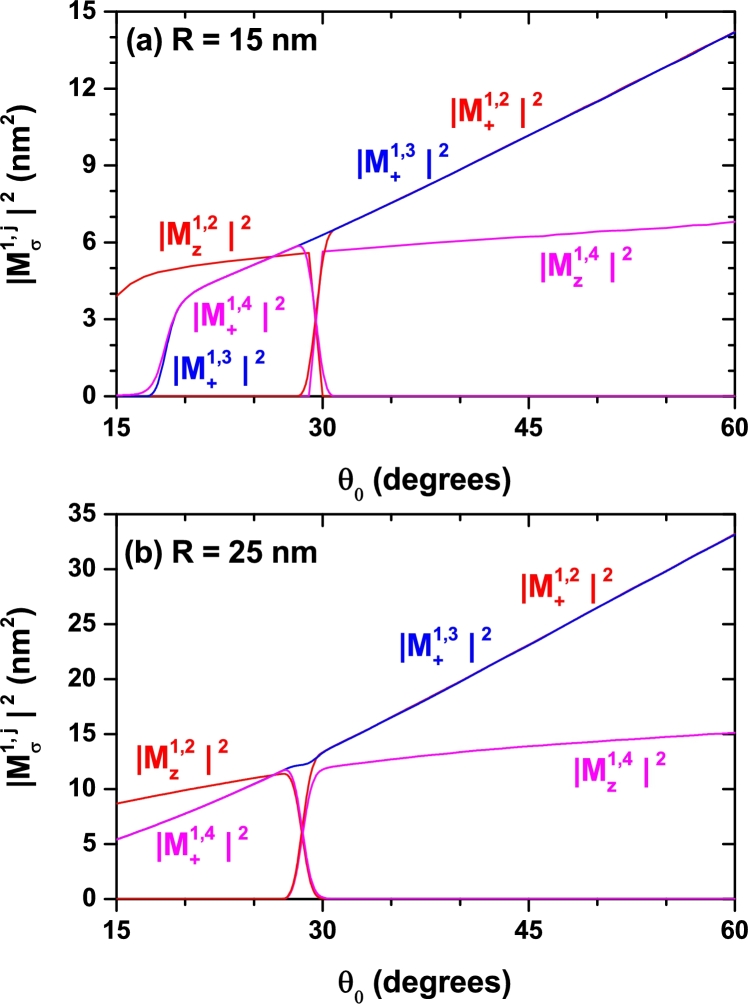
Square of the nondiagonal reduced dipole matrix elements from the ground state to the several lowest excited states (1 → *n*, with *n* = 2,3,4,…) for a confined electron in a spherical cone-shaped GaAs-Al_0.3_Ga_0.7_As quantum dot as a function of the apical angle of the structure. Calculations are for *z*-polarized (*ξ* = *z*) and circular-polarized (*ξ* = +) incident radiation. The results are for *z*_*i*_ = 0 with *R* = 15 nm (a) and *R* = 25 nm (b).

**Figure 10:**
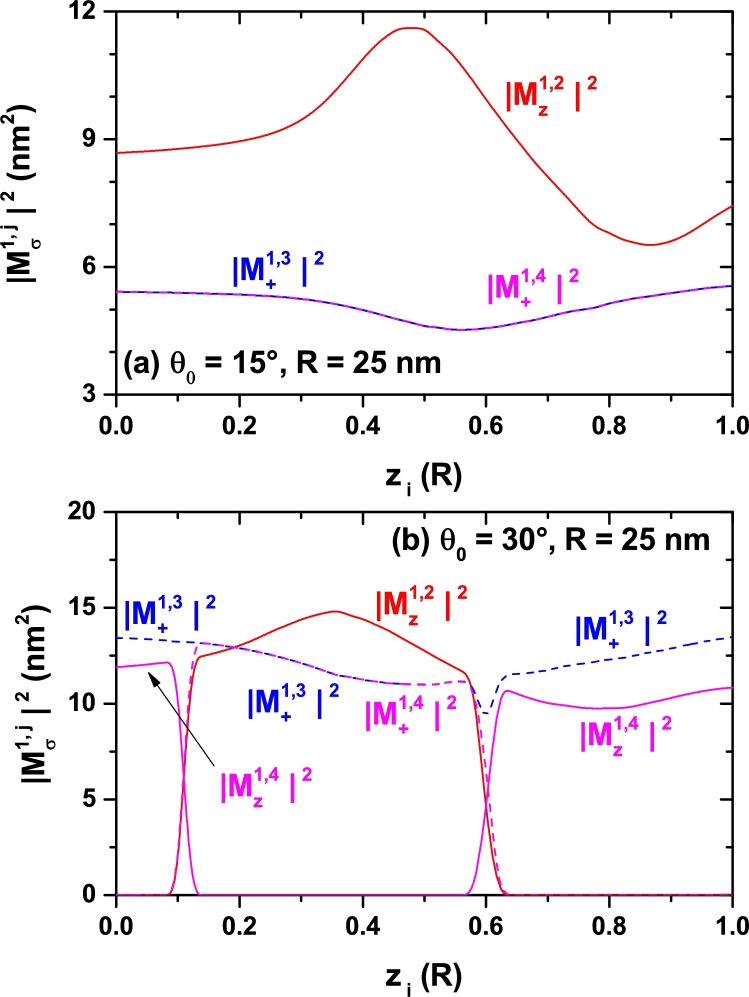
Square of the nondiagonal reduced dipole matrix elements from the ground state to the several lowest excited states (1 → *n*, with *n* = 2,3,4,…) for a confined electron in a spherical cone-shaped GaAs-Al_0.3_Ga_0.7_As quantum dot as a function of the impurity position along the *z*-axis. Calculations are for *z*-polarized (*ξ* = *z*) and circular-polarized (*ξ* = +) incident radiation. The results are for *R* = 25 nm with *θ*_0_ = 15° (a) and *θ*_0_ = 30° (b).

Studying the optical absorption response requires the evaluation of the electric dipole moment matrix elements for the involved inter-level transition. In the Figs. 8, 9, and 10 we present the calculated squared modulus of the reduced elements $M_{fi}={\overset{˜}{M}}_{fi}/e$. Each figure replicates the geometry setup chosen for Figs. 4, 5, and 7, respectively. Besides, two distinct polarizations for the incident light are assumed: a linear one oriented along the *z*-direction (parallel to the cone axis) and another, circular, oriented in the perpendicular plane. Since we are considering low temperatures, it is assumed that only the ground state is populated initially. Therefore, all transitions considered have it as the initial one, |i=1〉. The second superscript indicates, in each case, the final state. It is worth highlighting the situations observed in the Figs. 9 and 10 in which one may observe intervals of forbidden transitions for which the corresponding off-diagonal dipole moment contribution vanishes. This takes place in a small region around $\theta_{0}=30{}^{\circ}$ for fixed values of *R* and $z_{i}=0$ and within two ranges of $z_{i}$ values in the specific case of $\theta_{0}=30{}^{\circ}$ and R=25 nm. This kind of selection rule occurs due to symmetry reasons because in such cases the geometry of the SCS-QD confers a spatial dependence to the participating wave functions that produces zero values of the associated matrix elements. Let us analyze, for example, the way in which in Fig. 9(a), particularly at $\theta_{0}=30{}^{\circ}$, the change $|M_{z}^{1,2}{|}^{2}\to |M_{z}^{1,4}{|}^{2}$ is presented. Throughout the range of calculated angles, the $\Psi_{1}$ state has *s*-like symmetry. For $15{}^{\circ}<\theta_{0}<30{}^{\circ}$, the $\Psi_{2}$ state has $p_{z}$-like symmetry and the $\Psi_{3}$ and $\Psi_{4}$ states, which are degenerated, have $p_{x}$ and $p_{y}$ symmetry, respectively (see Fig. 6). For $30{}^{\circ}<\theta_{0}<60{}^{\circ}$ things are reversed, the $\Psi_{2}$ and $\Psi_{3}$ states, which are degenerate, have $p_{x}$ and $p_{y}$ symmetry, respectively, and the $\Psi_{4}$ state has $p_{z}$-like symmetry (see the Fig. 6 and its corresponding discussion). Given that for *z*-polarized radiation the matrix elements are only different of zero when the wave functions have simultaneously the same symmetry with respect to the x=0 and y=0 planes, this explains why only 1*s*-like → $p_{z}$-like transitions are allowed, giving the behavior shown in Fig. 9(a) for that matrix elements.

Here is the place where we must clarify the reason why in our studies we have proceeded to solve the full 3D problem of the differential equation, without making use of the azimuthal symmetry of the problem. Because in the study we have been interested in knowing the effects of incident radiation with linear polarization in the *z*-direction and circular polarization in the *xy*-plane, the particular situation of the circular polarization forces us to take into account the full distribution of the wave function. Solving the axis-symmetric problem does not provide direct information on how the wave functions behave when the azimuthal angle is swept, that is, there is no *xy*-description of the wave function. Therefore it is not possible to predict what they will be the results of the expected value of the dipole moment for polarized radiation along the *x*- and *y*-axes, which by combining at the end give rise to the circular polarization.

**Figure 11:**
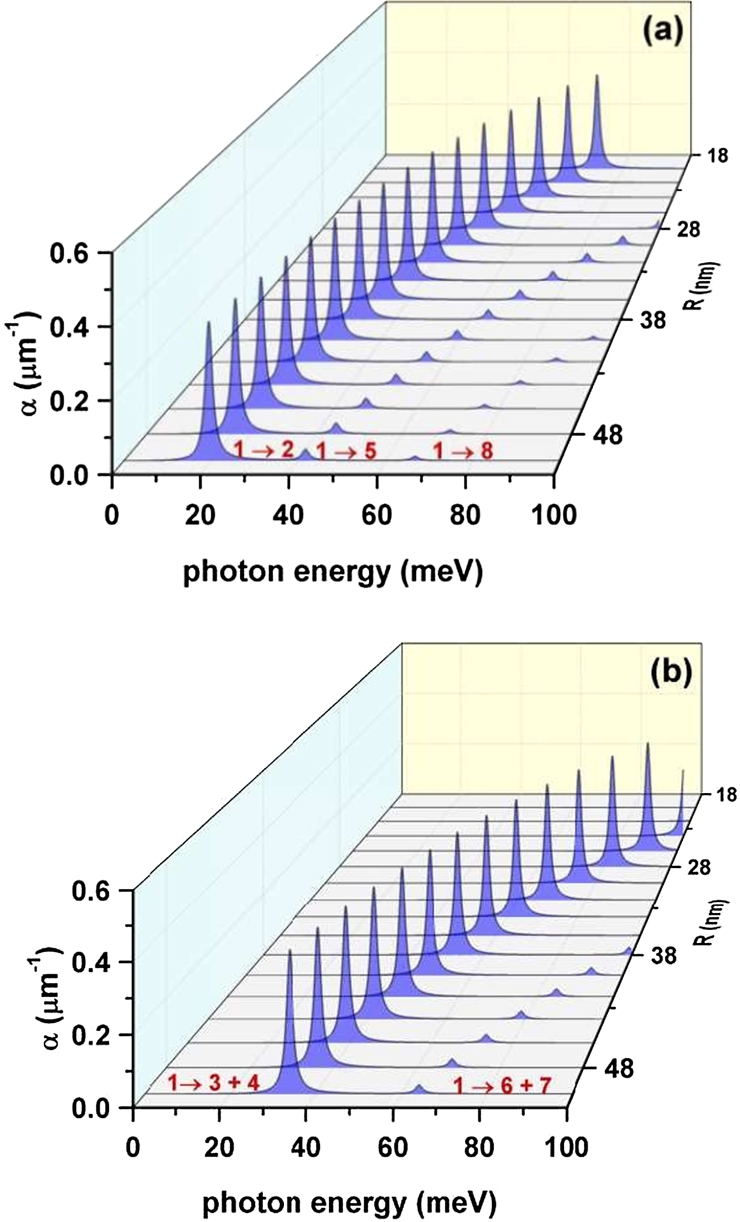
The optical absorption coefficient calculated as a function of the incident photon energy and the radius of the cone-shaped GaAs-Al_0.3_Ga_0.7_As quantum dot, with the data presented in the Figs. 4(a) and 8(a): for linear *z*-polarization (a) and for circular polarization of the resonant incident light (b).

The coefficient of optical absorption is then evaluated using Eq. (2) for intersubband transitions involving the results for the energy spectrum and dipole moment presented in Figs. 4(a) and 8(a), respectively, for the variation of the allowed energies and electric polarizations with respect to the size *R*. The choice of this setup is made in order to provide a particular case for illustration, since it is possible to present an analogous discussion for the optical response, based on the behavior of electron states as a consequence of the variation of the apical angle. The outcome of the calculation appears in Fig. 11(a) for the allowed transitions under *z*-polarization and in Fig. 11(b) for those under circular polarization of the incoming light. It is possible to identify in all cases the redshift of the optical response associated to the reduction of the inter-level energies caused by the increment in *R*, as noticed from Fig. 4(a). The amplitude of the most prominent absorption resonant peaks evolves in such a way that almost no change is apparent. The reason for this phenomenon lies in the quantitative compensation that arises when one multiplies the squared modulus of the dipole matrix elements, that is an increasing function of *R* by the resonant frequency which is, in fact, a decreasing function of the cone size.

**Figure 12:**
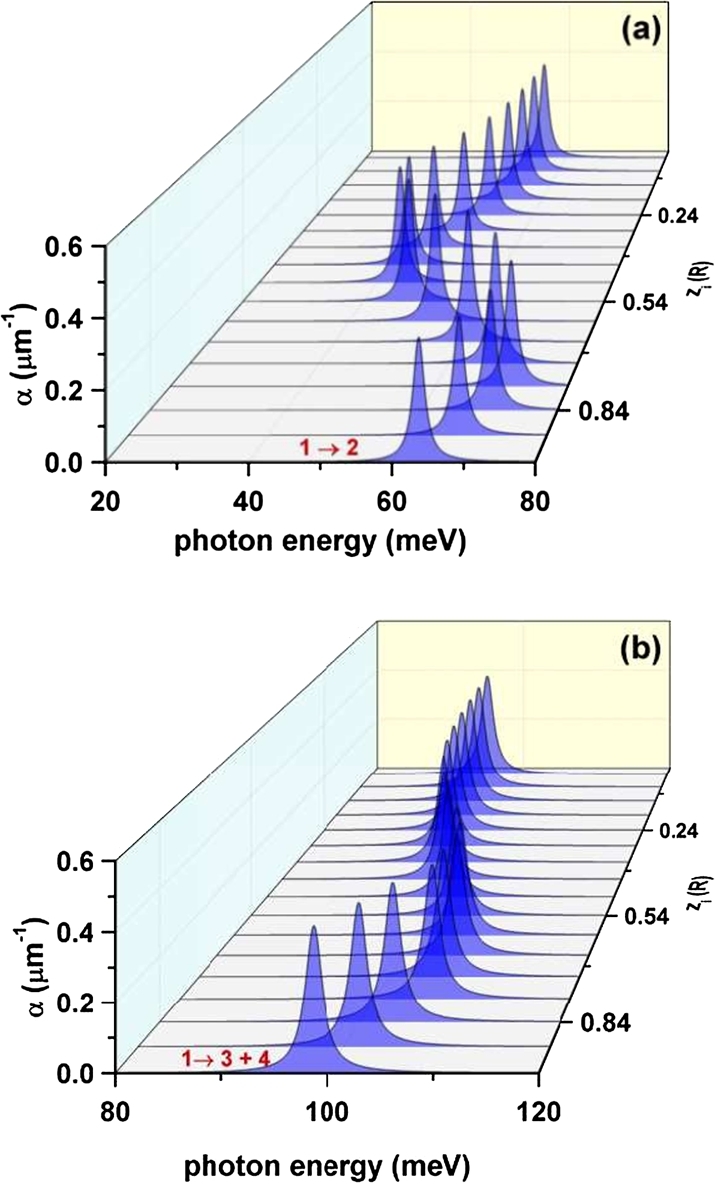
The optical absorption coefficient calculated as a function of the incident photon energy and the impurity position for a confined electron in a cone-shaped GaAs-Al_0.3_Ga_0.7_As quantum dot. The results are obtained with the data presented in the Figs. 7(a) and 10(a): for linear *z*-polarization (a) and for circular polarization of the resonant incident light (b).

In addition, the Fig. 12 contains the results of the calculated inter-level light absorption coefficient using as input data the results depicted in Figs. 7(a) and 10(a), for the electron energies and electric dipole moment matrix elements, respectively. In Fig. 12(a) we present the outcome corresponding to the allowed transitions under linear, *z*-oriented polarization of the incident electromagnetic radiation, whereas in the Fig. 12(b), the absorption response associated with incident light of circular polarization. The variation in the energy position of the resonant absorption peak is, again, governed by the behavior of the inter-level energy differences with respect to the variation in the axial position of the donor impurity atom. In consequence, one may notice a mixed shifting which is more pronounced in the case of linearly polarized incident light. That is, there is initially a slight redshift, then a noticeable blueshift takes place, followed by another displacement towards smaller frequencies. To just consider a specific example, the explanation for the presence of the blueshift in the interval above $z_{i}>0.5\;R$ of Fig. 12(a) can be found by noticing from Fig. 7(a) the increment in the separation between the ground and first excited levels that leads to the increment in the resonant transition energy difference. With regard to the resonant peak amplitude, the reader may observe that there is a very small variation along the range of $z_{i}$ considered. The explanation for that situation can be found in the same competition –above discussed– between the variations of both the inter-level energies and electric dipole matrix elements as functions of the donor impurity position.

### 3.1 A comparison with other theoretical and experimental reports

**Figure 13:**
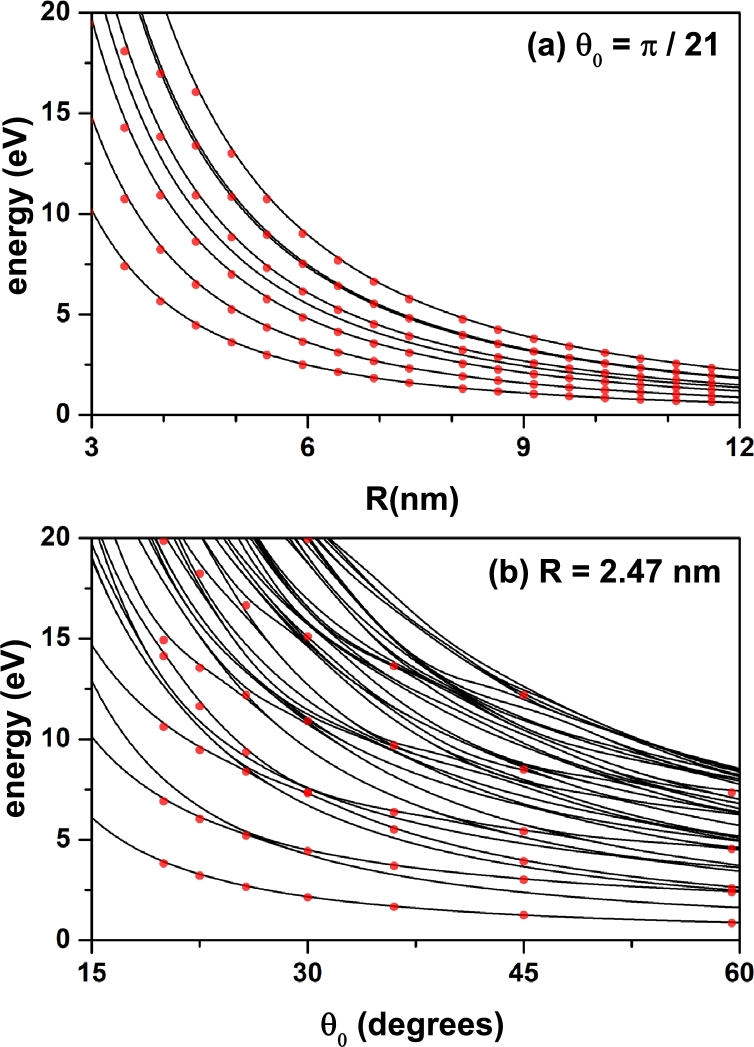
Energy of the lowest confined electron states in a spherical cone-shaped GaN quantum dot –with donor impurity located at the cone apex– as a function of the structure radius (a) and apical angle (b). The results are for *z*_*i*_ = 0 with *θ*_0_ = *π*/21 (a) and *R* = 2.47 nm (b). The lines are obtained via the numerical FEM calculations whereas the full symbols result from an analytical procedure (see the text).

**Figure 14:**
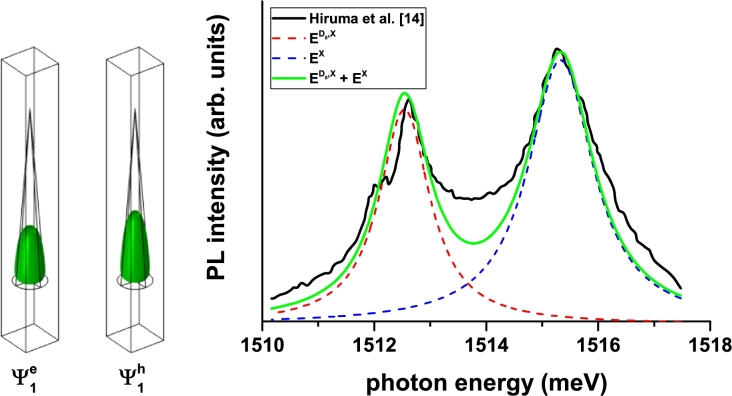
Photoluminescence energy transition for a confined electron-hole pair in spherical cone-shaped GaAs quantum dot. Theoretical findings are for heavy-hole exciton recombination (blue dashed line) and donor-impurity-heavy-hole recombination (red dashed line). The solid green line represents the mentioned two recombinations. The solid black line corresponds to the experimental findings by Hiruma et al. [14] for a GaAs truncated whiskers with hexagonal base. Also, the density of probability for the uncorrelated electron and heavy-hole are depicted. The parallelepiped contains the conical structure used to model the experimental structure.

In this subsection we proceed to make a comparison between the results obtained through the numerical method used in this article with some available theoretical and experimental reports. They appear in Figs. 13 and 14, respectively.

First, in Fig. 13 we present the energy of some electronic states in a spherical cone-shaped GaN QD with infinite confinement potential considering a donor impurity located at the apex of the cone. This is a very particular case where the problem has an analytical solution. Here, the Schrödinger equation in spherical coordinates is separable. The solution for the radial part of the wave function is described by a linear combination of Whittaker functions and the angular part results in a linear combination of Legendre polynomials; all subject to the appropriate boundary conditions to ensure that the wave function becomes zero on the surfaces of the cone (see above the subsection 2.1). The calculations have been made for $m_{e}^{⁎}=0.19\;m_{0}$ and $\varepsilon_{r}=8.9$, whereby the effective Bohr radius and the effective Rydberg are 2.47 nm and 32.6 meV, respectively. In Fig. 13, the lines correspond to our findings using the FEM technique while the solid symbols correspond to the results through the exact solution. As can be seen noticed, there is an excellent agreement between the two types of results which confirms the validity of the numerical FEM used in this work.

In Fig. 14 we show our results for the photoluminescence peak (PL-peak) energy position in a conical spherical-shaped GaAs QD surrounded by a matrix of ${\text{Al}}_{x}{\text{Ga}}_{1-x}\text{As}$. The experimental results are represented by the trembling line and correspond to those published in Ref. [14] where the system consists of GaAs truncated whiskers with hexagonal base. The authors report that the average dimensions of the structures are 150 nm and 20 nm in the major and minor diameters, respectively, with a total height of 1 μm. In Fig. 19 of the mentioned reference, the authors present two very well defined structures. The authors associate the first structure, which has an energy of 1515.4 meV, with a free exciton peak and the second, whose energy is 1512.5 meV, with a neutral carbon acceptor bound exciton peak.

Here, we have proceeded to simulate that study using our model of a cone-shaped GaAs QD. For that purpose, the dimensions of our structure are R=1000 nm and $\theta_{0}=5{}^{\circ}$, with which the radius of the cone base is 150 nm. The values employed for the effective mass and confinement potential parameters are $0.0665\;m_{0}$ and 262 meV ($0.45\;m_{0}$ and 174 meV) for the electron (for the heavy hole). The static dielectric constant is set at 13, corresponding to GaAs; since the carriers are essentially confined within the cone region. Besides, we have considered a donor impurity located at the cone gravity center.

Given that the cone can be considered as a convolution of many cylindrical structures whose average effective radius is 400 nm and following the works of Brown and Spector [34], [35], we have used a binding energy of 1 effective Rydberg (1 Ry = 4.737 meV) for the free exciton and 1.3 effective Rydberg (1 Ry = 5.43 meV) for the donor impurity. Our calculations yield 1512.54 meV for the PL-peak associated with the donor impurity and 1515.33 meV for the free exciton. As commented, the comparison between calculations and measurements from Ref. [14] appear in Fig. 14. We have considered a Lorentzian distribution of the PL-peak with a damping parameter of 0.3 meV for the donor impurity and 0.5 meV for the free exciton. As can be noticed, there is an excellent agreement between our findings and those of Hiruma et al. [14]. This fact confirms the interpretation related with the peak associated to a free exciton, but raises a controversy about the origin of the peak associated with impurities. While those authors associate it with acceptor impurities, our work concludes that, in order to exhibit a PL-peak at the reported energy value, the system has to be doped with donor impurities because that is, precisely, the kind of impurities the lowest energy peak matches with. The probability densities for the ground state of an electron and a heavy hole have also been presented in Fig. 14 confirming that the maximum is concentrated around the center of gravity of the cone and that the state of the hole is more extended in the space given its less effective Bohr radius.

## 4 Conclusions

In this work we have investigated the properties of the conduction band spectrum in spherical cone-shaped GaAs-Al_0.3_Ga_0.7_As quantum dots, with the influence of a donor impurity center. The effect of modifying the size and apical angle of the conical structure on the electron-impurity energy levels is particularly discussed. It is found that certain specific geometric and impurity-position configurations lead to forbidden inter-level transitions due to the particular symmetry of the associated wave functions. Taking into account those features, we calculated the coefficient of optical absorption due to the allowed transitions in two particular geometric setups. From such an evaluation it is possible to identify the behavior of the optical response under linearly and circularly polarized incident radiation, identifying very small changes in the resonant peak amplitudes and a progressive redshift of the curve when the cone size changes under fixed apical angle conditions. At the same time, for a given cone size (lateral and angular dimensions fixed) one may detect a mixed shifting to the red and the blue of the absorption coefficient, associated with the change in the impurity position along the cone axis.

Additionally, two comparison processes were performed in order to validate our theoretical procedure. The first relates with a particular situation in which a conical-spherical sector quantum dots exhibits a donor atom at the cone apex, with an additional infinite confinement potential at the dot surface. In this case, analytical solutions can be derived and numerical data on GaN structure have been produced. The agreement with the same calculation using our finite element scheme is excellent.

The second comparison involves experimental reports on photoluminescence peak energies in GaAs-based truncated-whisker-like quantum dots with hexagonal base. It is obtained a very good coincidence between calculated and measured results. Besides, we have been able to identify the impurity-related peak energy as one with donor center instead of acceptor one, as previously claimed. This could encourage to perform additional experiments on this kind of quantum dot structures. Up to our knowledge, this is the first time that a theoretical investigation is carried out in order to compare with those experimental results.

## Declarations

### Author contribution statement

M.E. Mora-Ramos: Analyzed and interpreted the data; Wrote the paper.

A. El Aouami, E. Feddi: Contributed analysis tools or data.

A. Radu: Conceived and designed the analysis; Wrote the paper.

R.L. Restrepo, J.A. Vinasco: Contributed analysis tools or data; Wrote the paper.

A.L. Morales: Conceived and designed the analysis.

C.A. Duque: Conceived and designed the analysis; Analyzed and interpreted the data; Contributed analysis tools or data; Wrote the paper.

### Funding statement

This work was supported by Mexican CONACYT (Grant CB-2017-2018 No. A1-S-8218); Colombian Agencies: CODI-Universidad de Antioquia (Estrategia de Sostenibilidad de la Universidad de Antioquia and projects “Propiedades magneto-ópticas y óptica no lineal en super redes de Grafeno” and “Estudio de propiedades ópticas en sistemas semiconductores de dimensiones nanoscópicas”) and Facultad de Ciencias Exactas y Naturales-Universidad de Antioquia (CADexclusive dedication project 2019-2020); and el Patrimonio Autónomo Fondo Nacional de Financiamiento para la Ciencia, la Tecnología y la Innovación Francisco José de Caldas (project: CD 111580863338, CT FP80740-173-2019). M.E. Mora-Ramos, A. Radu, A.L. Morales, and C.A. Duque were supported by the Universidad EIA and Universidad de Antioquia (project “Propiedades optoelectrónicas en puntos cuánticos semiconductores”).

### Competing interest statement

The authors declare no conflict of interest.

### Additional information

No additional information is available for this paper.
